# Setting sodium targets for pre-packaged foods in China — an exploratory study

**DOI:** 10.3389/fnut.2023.1231979

**Published:** 2023-11-01

**Authors:** Puhong Zhang, Jiguo Zhang, Emalie Rosewarne, Yuan Li, Le Dong, Feng J. He, Mhairi Brown, Simone Pettigrew, Rain Yamamoto, Chizuru Nishida, Aidong Liu, Xiaoguang Yang, Bing Zhang, Gangqiang Ding, Huijun Wang

**Affiliations:** ^1^The George Institute for Global Health, Beijing, China; ^2^Faculty of Medicine, The George Institute for Global Health, University of New South Wales, Sydney, NSW, Australia; ^3^Key Laboratory of Trace Element Nutrition of National Health Commission, National Institute for Nutrition and Health, Chinese Centre for Disease Control and Prevention, Beijing, China; ^4^Wolfson Institute of Population Health, Barts and The London School of Medicine and Dentistry, Queen Mary University of London, London, United Kingdom; ^5^Standards and Scientific Advice on Food and Nutrition Unit, Department of Nutrition and Food Safety, World Health Organization, Geneva, Switzerland; ^6^Safe, Healthy and Sustainable Diet Unit, Department of Nutrition and Food Safety, World Health Organization, Geneva, Switzerland; ^7^China National Center for Food Safety Risk Assessment, Beijing, China

**Keywords:** sodium, benchmarks, exploratory study, China, pre-packaged foods

## Abstract

**Introduction:**

Setting sodium targets for pre-packaged food has been a priority strategy for reducing population sodium intake. This study aims to explore the attitudes and considerations of researchers and key stakeholders toward implementing such policy in China.

**Methods:**

An exploratory study comprising a survey and a focus group discussion was conducted among 27 purposively selected participants including 12 researchers, 5 consumers, 4 administrators, 3 industry association representatives and 3 food producers. The survey/discussion covered the key questions considered when developing/promoting sodium targets. Free-text responses were manually classified and summarized using thematic analysis.

**Results:**

Two-thirds of the participants supported target-setting policy. Researchers and administrators were most supportive, and food producers and associations were least supportive. Adapted WHO food categorization framework was well accepted to underpin target-setting to ensure international comparability and applicability for Chinese products. Maximum values were the most agreed target type. The WHO benchmarks were thought to be too ambitious to be feasible given the current food supply in China but can be regarded as long-term goals. Initially, a reduction of sodium content by 20% was mostly accepted to guide the development of maximum targets. Other recommendations included implementing a comprehensive strategy, strengthening research, engaging social resources, establishing a systematic monitoring/incentive system, maintaining a fair competitive environment, and developing a supportive information system. Target-setting policy was acceptable by most stakeholders and should be implemented alongside strategies to reduce discretionary salt use.

**Discussion:**

Our findings provide detailed guidance for the Chinese government when developing a target-setting strategy. The methods and results of this study also provide meaningful references for other countries to set sodium targets for pre-packaged foods and implement other salt reduction strategies simultaneously.

## Introduction

Salt/sodium reduction is recognized as one of the most cost-effective interventions to reduce the burden of chronic noncommunicable diseases. As highlighted in the WHO SHAKE package, population-wide salt reduction requires comprehensive strategies (1). A key strategy is harnessing the food industry to use less salt through food reformulation. Establishing sodium targets or target-setting has been identified as a priority strategy to promote food reformulation due to its scalability and sustainability and large potential benefit (2). Per the recent WHO global report on sodium intake reduction, however, only 65 countries around the world have implemented this policy (3).

To accelerate progress, the WHO established global sodium benchmarks for 11 categories and 58 subcategories of foods in 2021 to support countries to introduce national target-setting policies. The benchmarks should be achievable goals because they have been determined as the lowest maximum value of existing national or regional targets for each subcategory (4). However, gradually reduced sodium targets are acceptable for most countries especially for those where the pre-packaged foods have relatively high baseline sodium concentrations and the dietary sodium is mainly from cooking/table salt.

China is among the countries with the highest population salt intake, with the estimated salt intake for adults at around 11 g/d in 2020 (5). Over 80% of sodium intake comes from salt and condiments added during cooking rather than from pre-packaged food (6, 7). To achieve the national goal of 20% reduction in salt intake by 2030 (8), China has launched several national programs to reduce the discretionary salt use through mass media campaigns and environment development (9–13). Regarding salt reduction through pre-packaged foods, except for mandatory labeling of sodium (14), no target-setting policy has been released by the government. This is despite the fact that the consumption of pre-packaged foods in China is increasing (15, 16), and most pre-packaged foods contain more sodium than their counterparts in other countries (17–19). Till now, only a set of voluntary sodium targets has been included in the Guideline for Salt Reduction for Chinese Food Industry issued by a research group in 2019 (20).

Around the world, there has been substantial experience in developing and implementing sodium targets for different foods. However, the development and implementation of sodium targets are complex, and each country needs to consider their own conditions when determining the leading institutes or organizations, type of targets (maximum or mean), approaches used to develop/update the targets, priority foods, type of implementation (voluntary or mandatory), and systematic monitoring and evaluation.

To drive progress on sodium reduction in pre-packaged foods in China, two studies were undertaken in parallel: (1) a quantitative study modeling the effect of different sodium target designs on sodium reduction to support the selection of the initial targets by government (21), and (2) the current exploratory study (mainly based on qualitative interview) to find out the opinions and suggestions on how to initiate such strategy by interviewing experienced researchers, policy-makers, delegates of relevant social organizations, and influential representatives from companies and consumers in China.

For specific, the purpose of the exploratory study is to explore (1) general attitudes toward a target-setting policy, (2) attitudes toward adopting the WHO benchmarks, (3) key parameters for initiation, and (4) opportunities and challenges\barriers to be addressed when starting a target-setting policy in China.

## Methods

### Study context

The National Institute for Nutrition and Health (NINH) and The George Institute for Global Health (TGI) China co-led the work based on their collaboration in the Action on Salt China (ASC) program (22). A working group was established in June 2021 to explore how to leverage existing evidence and the WHO benchmarks to facilitate the Chinese government setting sodium targets for pre-packaged foods. Several virtual technical consultation meetings were convened from July to December 2021 to refine the study design. An expert committee, composed of 5 national nutrition experts from NINH, TGI, WHO China office, Chinese Nutrition Society and Peking University, was also established to review the protocols and help nominate the participants. All the consultants and experts have direct experience in setting sodium targets or understanding of the technological aspects of sodium use and sodium reduction.

### Participants and recruitment

Thirty potential participants were nominated by the expert committee. They came from governments, research organizations, social organizations, food enterprises and consumers, and had recognized expertise and significant influence in changing food supply through policymaking/administration, research, management, reformulation/development, and purchasing/consumption. Consumer representatives mainly came from fields such as media, advertising, and marketing, mainly considering their representativeness and influence on public opinion. All participants were contacted via email and 27 of them agreed to participate.

### Data collection

The is a mixed method study, consisted of a structured individual survey and a focus group discussion. It was conducted from January to March 2022. Both the survey and group discussion were guided with the same tool (Appendix S1: Guide and questionnaire for the survey and group discussion) which covered the key questions considered by countries and the WHO when developing and promoting sodium targets/benchmarks. It contained 13 questions, of which the first 10 questions were single/multiple-choice questions accompanied by open areas to collect additional opinions on certain topics. The last 3 questions were open-ended.

The individual survey was delivered through Questionnaire Star,^1^ a widely used electronic survey platform. People could use their computers or mobile phones to complete the survey. To enable the participants to make informative choices and comments, a 1.5-h systematic background introduction was given in advance which covered the global experience of target-setting for salt reduction, the WHO global sodium benchmarks for different food categories, the key findings of the parallel quantitative study, and the current strategies for salt reduction in China. The individual survey was guided by an experienced investigator, and the participants were encouraged to enter their answers and opinions directly into the electronic questionnaire platform without discussion with others.

Two focus group discussions were convened after the individual survey: onsite group and online group. The online group was organized for those unable to attend the face-to-face discussion due to COVID-19. All the participants were encouraged to express their opinions and suggestions which were recorded in the open text areas of the outline which had been printed out in advance for each participant. Near the end of the group discussion, all the participants were told the main findings of the individual survey with the purpose of helping the participants further refine their thoughts. The same investigator moderated the whole process following the guide and structured questionnaire.

### Data analysis

Participants were characterized according to their role and organization. Responses to the closed survey questions were entered into Excel (Microsoft 365 Apps for enterprise), analyzed and summarized. The free-text responses for each question were manually classified and summarized by the core research team following Braun and Clarke’s method of thematic analysis (23). This includes becoming familiarized with the data, developing initial codes, collating these codes to identify emerging themes, and then ensuring these themes connect to the data by creating a thematic map. The responses to the last question “13. Are there any other considerations?” were all re-allocated into the other 12 questions because no new theme was found. When reporting the results, the questions were re-organized to fit the four study objectives.

## Results

All 27 participants completed the survey and focus group discussion. Of these, 8 participated online group discussion due to COVID-19. By role, there were 12 researchers from nutrition or food fields (5 from national institutes, 3 from universities, 3 from international institutes, and 1 from a private institute), 5 consumers (2 with education background in health and 3 without), 4 administrators (1 from government, 3 from government agencies), 3 senior delegates from professional associations (food industry, condiments, and meat), and 3 representatives of food producers (2 international and 1 local) (Table 1).

**Table 1:** The code and characteristics of the participants.

Code	Gender	Age	Major role
Adm 1	Female	18–44	Administrator
Adm 2	Female	18–44	Administrator
Adm 3	Female	18–44	Administrator
Adm 4	Male	45–59	Administrator
Ass 1	Female	18–44	Association delegate
Ass 2	Female	18–44	Association delegate
Ass 3	Male	18–44	Association delegate
Cons 1	Female	18–44	Consumer
Cons 2	Female	45–59	Consumer
Cons 3	Female	45–59	Consumer
Cons 4	Male	18–44	Consumer
Cons 5	Male	18–44	Consumer
FP 1	Female	18–44	Food producer delegate
FP 2	Female	18–44	Food producer delegate
FP 3	Female	45–59	Food producer delegate
Res 1	Female	18–44	Researcher
Res 2	Female	18–44	Researcher
Res 3	Female	18–44	Researcher
Res 4	Female	45–59	Researcher
Res 5	Female	45–59	Researcher
Res 6	Male	18–44	Researcher
Res 7	Male	18–44	Researcher
Res 8	Male	18–44	Researcher
Res 9	Male	45–59	Researcher
Res 10	Male	45–59	Researcher
Res 11	Male	45–59	Researcher
Res 12	Male	> = 60	Researcher

Responses to the first 10 questions with closed choices are presented in Table 2. The major findings from the open-ended questions are summarized in Tables 3, 4, including illustrative quotes organized according to the four research objectives.

**Table 2:** Results of the interview based on the closed questions.

Questions and choices	*n*	Proportion (%)	By stakeholder group (n/N)
1. [General view] toward the target-setting strategy			
(1) Necessary to set as soon as possible because the salt content of processed foods in China is too high, and the consumption is increasing.	18	66.7	Res (11/12); Adm (4/4); Cons (3/5)
(2) Unnecessary because the contribution of processed food to sodium intake in China is not high (around 30%, including condiments like soy sauce and fish sauce), and therefore it is not cost-effective.	9	33.3	FP (3/3); Ass (3/3); Cons (2/5); Res (1/12)
2. [Enterprise’s attitude] toward the target-setting strategy			
(1) In any case, the enterprises will not be willing to cooperate.	1	3.7	Cons (1/5)
(2) Enterprises may actively cooperate if they find the sodium contents of some products are too high and might be doing harm to the sales.	15	55.6	Res (9/12); Ass (2/3); Adm (2/4); FP (1/3)
(3) Others	11	40.7	Cons (3/5); Res (3/12); FP (2/3); Adm (2/4); Ass (1/3)
3. [General opinions on WHO benchmarks]			
(1) The WHO benchmarks draw on the experience of many countries and could be adopted directly.	1	3.7	Res (1/12)
(2) It can be used for reference but must be adjusted according to China’s national conditions.	25	92.6	Res (11/12); Adm (4/4); Cons (4/5); FP (3/3); Ass (3/3)
(3) It is not necessary or impossible to refer to the WHO sodium benchmarks.	1	3.7	Cons (1/5)
4. [Type of targets] you prefer for each category of food			
(1) Only setting the maximum sodium targets	13	48.1	Res (8/12); FP (2/3); Cons (2/5); Adm (1/4)
(2) Setting both the maximum and average sodium targets	11	40.7	Res (4/12); Adm (3/4); Cons (3/5); Ass (1/3)
(3) Others	3	11.1	Ass (2/3); FP (1/3)
5. [Maximum target setting approaches] for a certain category of food			
(1) Adopting the WHO sodium benchmarks directly	1	3.7	Res (1/12)
(2) Adopting a percentile level of sodium content such as the 90th or 75th percentile level	18	66.7	Res (10/12); Cons (5/5); Adm (3/4)
(3) Other methods	8	29.6	FP (3/3); Ass (3/3); Res (2/12)
6. [Average target setting approaches] for a certain category of food			
(0) Participants who skipped this question	6	22.2	FP (3/3); Ass (2/3); Res (1/12)
(1) A simple average target	1	3.7	Cons (1/5)
(2) An average target weighted by sales or consumption of foods in the category	20	74.1	Res (11/12); Adm (4/4); Cons (4/5); Ass (1/3)
7. Use of WHO [food categorization framework] for global sodium benchmarks			
(1) Should be copied because it is specially established for global sodium benchmarks, reasonable and conducive to international comparison	1	3.7	Cons (1/5)
(2) It should be referred to as far as possible but needs to be supplemented appropriately according to the advantageous products in China.	17	63.0	Res (10/12); Adm (4/4); Cons (3/5)
(3) The WHO system is not suitable for China, so other classification systems should be selected.	8	29.6	FP (3/3); Ass (2/3); Res (2/12); Cons (1/5)
(4) I am not an expert in this field and cannot judge which food categorization system is the best.	1	3.7	Ass (1/3)
8. [Priority food categories] to set sodium targets for at the initial stage			
(1) Based on the average sodium levels of individual food categories, the higher ones are preferred.	0	0.0	
(2) Based on the average sodium levels of individual food categories weighted by their sales/consumption shares, the higher ones are preferred.	20	74.1	Res (8/12); Cons (5/5); Adm (3/4); FP (2/3); Ass (2/3)
(3) Setting sodium targets for all kinds of food according to certain rules, and there is no need to choose priority food categories.	4	14.8	Res (2/12); FP (1/3); Adm (1/4)
(4) Others	3	11.1	Res (2/12); Ass (1/3)
9. [Mandatory or voluntary] if target-setting strategy will be implemented			
(1) Voluntary	11	40.7	Res (4/12); FP (2/3); Ass (2/3); Cons (2/5); Adm (1/4)
(2) Voluntary first, and transition to mandatory for appropriate categories	12	44.4	Res (7/12); Adm (2/4); Ass (1/3); FP (1/3); Cons (1/5)
(3) Mandatory	4	14.8	Cons (2/3); Adm (1/4); Res (1/12)
10. [Principle of timeline setting]			
(1) No need to set an exact timetable because processed food is not the main source of sodium intake.	5	18.5	FP (2/3); Ass (2/3); Cons (1/5)
(2) Should be consistent with the progress of “reducing salt by 20% by 2030.”	15	55.6	Res (9/12); Adm (3/4); Cons (3/5)
(3) Should be more radical because the sodium targets might be voluntary and set for only some products	5	18.5	Res (3/12); Adm (1/4); Cons (1/5)
(4) Others	2	7.4	Ass (1/3); FP (1/3)

Res, researcher; FP, food producer; Adm, administrator from government or government agencies; Ass, representatives from national professional associations of food industry and specific foods which are national and industrial non-profit social organizations with close contact with the corresponding food enterprises to promote the sustainable, stable, and coordinated development of the food industry; Cons: consumer.

**Table 3:** Additional key opinions to the closed options of question 1–10 regarding research objective 1–3.

Objectives	Questions	Major opinions in form of quotes under different [themes]
1. General attitudes toward the target-sitting policy		
	Q1 General view about the target-setting strategy	
		[Opinions in favor of the policy] Adm 1: “Sodium reduction has been emphasized in the Healthy China 2030 Action. Setting sodium targets for pre-packaged foods will be a signal that can reflect the importance of salt reduction to the people and country and will be also a support for sodium reduction in other fields.” Adm 2: “The consumption of processed food of the younger generation is growing rapidly. From the perspective of prevention, sodium targets should be set; Moreover, the target-setting work should be carried out as soon as possible, because it may take a long time from formulation to effectiveness.” Res 3: “The establishment of sodium targets is conducive to guiding the development of salt reduction technology in food industry and improving consumers’ awareness of salt reduction.” [Opinions against the policy] Ass 1,2,3 and FP 1: “In-depth research and investigations should be done to identify its impact to food production, transportation and sales before implementing the policy.” Ass 2: “The food industry has been reducing sodium spontaneously, and there are many low sodium products on the market already, it is not necessary to set sodium targets.” Ass 2: “The food itself is not good or bad. Consumers can have a variety of choices for food taste. The key is educating consumers to select low-sodium products.”
	Q2 Enterprise’s attitude toward the target-setting strategy	
		[Positive opinions] Res 1,4,5,8, Adm 2 and FP 2: “Food manufacturers may have realized that the development of healthy food meets the people’s health needs, national policy requirements and corporate social responsibility, which is conducive to improving the company’s market image and sales.” [Neutral or negative opinions] FP 3, Cons 1 and Res 10: “Whether food manufacturers will take measures mainly depends on the acceptance of consumers and the attitude of the government.” Cons 2,3, Res 11 and Adm 1, FP 1: “The enterprise scale, product type, process conditions, storage and transportation mode, research and development ability and other factors affect the attitude and behavior of the enterprise. The enterprise may have low enthusiasm or even objection due to various difficulties. Therefore, when setting sodium targets, we need to comprehensively consider various situations, formulate reasonable targets and implementation plans, and pay attention to the accumulation of relevant technologies and enterprise education.”
2. Attitudes toward the WHO global sodium benchmarks		
	Q3 General opinions on WHO benchmarks	
		[In favor of direct use] Res 1: “The benchmarks could be copied because it is developed based on global experience, and direct use can improve the comparability of salt reduction effect with other countries.” [In favor of phased targets] Cons 2: “The WHO benchmarks are not applicable to China. What we can refer to and learn from is its ideas and methods” Res 3,4,6,9,10,11, Adm 1, 2 and Cons 1: “Most of the WHO benchmarks might be too radical for initial use. To be feasible, the targets should be set based on China’s national conditions including the baseline sodium contents and contribution to sodium intake of individual food categories.” Adm 1: “Considering that most Chinese products have much higher sodium content than the WHO benchmarks, we can take the benchmarks as the goal, but should take it step by step. At the same time, we should note that products in a few subcategories have reached the WHO benchmarks.”
	Q5 Approaches used to develop maximum targets	
		[Adopting the WHO benchmarks directly] (Only Res 1) Res 1: “It can improve the comparability of salt reduction effect with other countries.” [In favor of the percentile targets] Res 3,10 and Cons 3: “The percentile method is simple, and should be more appropriate to the national conditions” Res 9,11: “With continuous decrease of the sodium content of food products, the maximum targets determined by a specific percentile will also decrease, which will lead to a continuously lowered target.” [Challenging both] FP 2 and Ass 3: “Simply setting targets according to a certain approach is not scientific enough, and technical feasibility should be considered.” FP1,3 and Adm 3: “Product sales or their contributions to population sodium intake should also be considered.”
	Q7 Food categorization framework	
		[In favor or WHO categorization framework] Res 1: “It is recommended to directly copy the framework because it was established by WHO specifically for setting the maximum sodium targets for different foods based on experience from many countries and should be relatively reasonable. [Preferring other food categorization framework] FP 2,3, Ass 2 and Res 3: “The existing China food categorization system should be used, such as the China Food Categorization Framework for Food Production License.” FP 1, Ass 3 and Cons 2: “Due to different taste and eating habits between China and the west, there are great differences in dominating food products and the formulations of same products. China’s own categorization system should be developed.”
3. Key parameters for target-setting		
	Q4 Type of targets	
		[In favor of proposed options] Res 10: “The maximum method is easier to understand and more convenient for supervision.” Ass 1: “Using average levels at the same time can constrain the overall use of salt.” [Against setting maximum targets] FP 1: “The most important thing is to publicize and educate consumers. Food manufacturers provide diversified products, and it is suggested to comprehensively consider the enterprise’s product lines and not set a one-size-fits-all target for all products in a food category.”
	Q5 Approaches used to develop maximum targets (as above for Q5)	
	Q6 Approaches used to develop the mean targets (no additional comments)	
	Q7 Food categorization framework (as above for Q7)	
	Q8 Priority food categories	
		[Opinions in support of targeting priority foods using consumption/sales data] Adm 1–3, Cons 1–4, Res 2,4,5,12 and FP 1,2: “Consumption/sales data can help target major sodium intake contributors.” Res 5: “The academics can set targets for all food categories, and the government can start with priority foods.” [Opinions against targeting priority foods] Ass 3: “It is too early to talk about this. Production process and technical feasibility should be the basis.”
	Q9 Mandatory or voluntary	
		[Arguments supporting voluntary implementation] Ass 3, FP 3 and Res 5: “There is no reason to enforce the implementation of such policy. Sodium reduction is not an acute safety problem. The enterprise has the right to independently determine the salt contents of their products.” Res 4,7,9, Adm 2 and Cons 4: “In the case that the effect and impact of target-setting are not very clear, it is better to start the implementation voluntarily.” FP1, Adm 3, Ass 2, Res 3 and Cons 2: “Mandatory implementation may not work and may have a negative impact on industrial development. The key is to increase consumers’ awareness and provide enterprises with technical support and policy incentives, which may finally help enterprises gradually form a consensus on sodium reduction.” R7: “Mandatory implementation needs systematic monitoring and evaluation. The government is not ready yet.” Res 6,8,11, Adm 2 and FP 2: “The policy can cover all kinds of food, start voluntarily, and from less to more, gradually force all products to meet the targets. This can give enterprises a process of adaptation.” [Arguments supporting mandatory implementation] Cons 1: “If implemented voluntarily, the supervision may not be in place, and the policy may become mere formality” Res 10, Adm 1 and Cons 3: “Enterprises, especially small and medium-sized ones, will not actively cooperate with voluntary policy. Only mandatory implementation works.”
	Q10 Principle of timeline setting	
		[Agree with timeframe aligned with national goal] Adm 2, Res 1,5,9 and Cons 3,4: “The national goal is the most likely progress in salt reduction. Salt reduction in pre-packaged foods should not progress too quickly, otherwise people and businesses will not be able to accept it. A 20% salt reduction, completed within a few years, should be feasible. You can set this goal first, and then move down if it goes smoothly.” [Objection to setting a timeframe] Ass 3 and FP 1: “Unable to determine, because the targets have not been determined yet, and the 20% sodium reduction goal is measured by the national dietary sodium intake, which may not be suitable for certain foods.” Ass 1,2, FP 2,3 and Cons 2: “There is no need to set an exact timetable because pre-packaged food is not the main source of sodium intake, it just needs to keep up with the decline of discretionary salt use.” [Preferring a more aggressive timeframe] Res 6,8,9, Adm 1 and Cons 1: “The timeline should be more aggressive considering that China’s target-setting policy has fallen behind and is likely to be started voluntarily.”

**Table 4:** Key responses to question 11 and 12 regarding research objective 4 – opportunities and challenges\barriers to be addressed.

Major themes	[Sub-themes] and major quotes
(1) Policy	
	[Opportunities – Government is powerful] Res 1,4,5,9, FP 2,3, Ass 1 and Cons 1: “Our government has strong executive power.” Res 4: “Once the government determines to do so, it will be highly efficient and effective.” Res 7,8 and Adm 1: “The implementation of such strategy needs strong policy support and perfect regulatory system to ensure its effective implementation.” [Opportunities – Established policy environment] Res 6,8, Cons 2,3, Adm 2: “Salt reduction has been included in the National Healthy Lifestyle Action for All since 2007 and Healthy China 2030 in 2016.” Adm 2: “The China Nutrition Society has initially put forward a proposal of sodium targets in its guideline of salt reduction for food industry issued in 2021.” [Challenges or barriers to be addressed – Further policy advocacy] Res 4,9: “The government still has concerns about the impact of target-setting policy on health and other aspects. More government mobilization and communication are needed.” Res 1,4: “Sodium reduction in pre-packaged food involves multiple departments and industries. It seems that the government has not made up its mind and put forward a specific plan to coordinate all parties to form a joint force to promote sodium reduction.” [Challenges or barriers to be addressed – Needing synchronized implementation of comprehensive strategies] FP 1,3: “Family and dining out play a decisive role in sodium intake. The effect of reducing sodium intake through pre-packaged food is relatively low. Simultaneous implementation of comprehensive measures is important.” Res 10, Cons 2, Adm 3 and Ass 2: “The improvement of consumers’ awareness and the gradual change of taste for salt are the most important. The sodium reduction in pre-packaged food needs to be synchronized with other strategies targeting discretionary salt use.” [Challenges or barriers to be addressed – Strengthening research] Ass2: “At present, the conditions for establishing the corresponding sodium targets in China are not met, because there is still a lack of sufficient research and industry investigation.” Res 4,6,11, Ass 2 and FP3: “Food sodium reduction may lead to changes in entire supply chain of production and will also lead to changes in costs. All these need research and investigations to find out new technologies or solutions to adapt to these changes.” [Challenges or barriers to be addressed – Maintaining fair competitive environment] FP 1,3, Ass 3 and Res 8: “The implementation of the policy may affect the competitiveness of some products, especially for traditional high-sodium products like condiments.” Res 11: “The sodium content of international products is relatively low. China should consider the impact on domestic products when setting sodium targets or black-and-white lists. The policy implementation should be gradual and not rushed.” [Challenges or barriers to be addressed – Engaging social resources] Adm 1, Res 8, Cons 1: “At present, this strategy is only promoted by a few institutions and experts, which is weak and lacks sufficient influence. It requires the joint participation of all stakeholders and relevant social resources to form a joint force.” Res 9: “In terms of mobilizing social forces, the support of the government and policies is critical, but now it is not enough.” [Challenges or barriers to be addressed – Establishing effective performance appraisal and incentive system] Res 3,8,11 and Cons 3: “We should establish incentive mechanisms and policies. Subsidies, commendations, and publicity could be given to enterprises with good performance.” Adm 1,2 and Res 10: “How to attract and motivate enterprises to comply is the most difficult and critical. This requires top-level design to engage the key departments.” Res 9: “Third-party institutions and individuals should be encouraged to participate in the monitoring & evaluation.” Res 2,8,10,11 “It could be a black-and-white list or ranking system open to the public in real time, or to sign responsibility letters with the enterprises.” [Challenges or barriers to be addressed – Support of information system] Res 9: “A national pre-packaged food information platform should be established to support enterprises to check the level of sodium content of their food products among the same food categories and help them to judge whether their products have exceeded the maximum targets and determine whether they should reformulate their products and the degree of sodium reduction.”
(2) Social resources	
	[Opportunities] Res 4: “Various societies and associations in nutrition, food science, food industry, health education and promotion are core social resources which can contribute to the implementation of target-setting policy through training, research, and routine communication and organization.” [Challenges or barriers to be addressed] Res 6: “Most social forces, especially the food industry associations, have not played an active role in reducing sodium content of pre-packaged foods.” Adm 2, Res 9, FP 1,3 and Cons 1: “The government should clarify the responsibilities of various social forces, promote and support them to play their industry leadership, and lead member enterprises to reduce sodium through food reformulation.” Cons 4, Res11, Adm 2, Ass 1: “It is necessary to further promote the awareness of sodium reduction among the people and all social forces through public opinion guidance.”
(3) Technical aspects	
	[Opportunities] Res 4,9: “The salt content of most pre-packaged foods in China, especially condiments, processed meat and instant food products, is generally higher than that of other countries, and there is much room for sodium reduction.” Res 8, Adm1, Res 7: “Some scientific research institutions and enterprises have been exploring the improvement of food formula and have accumulated certain technology, but still need to be improved and promoted.” [Challenges or barriers to be addressed – Food reformulation] Ass1, Res 6 and Cons 2: “Sodium reduction and corresponding process improvement may lead to significant changes in product properties, food safety, packaging, storage and transportation methods, and will also increase costs.” FP 1: “If consumer tastes remain unchanged, it is difficult to reduce sodium for foods that rely on seasoning. It is a great challenge for the industry to reduce sodium even by 5%. Once mass production, it may have a great impact on enterprises.” Ass 2 and Res 3: “Some products need more salt or sodium additives. There are still unsolvable technical problems in reducing sodium content significantly.” Res 10: “When using low sodium sauces for cooking, people are likely to increase salinity by adding more. So, it is not necessary to reduce salt use for high sodium condiments, instead, we should educate consumers to use less.” FP 1: “As an alternative solution or a supplement to target-setting, food manufacturers could supply diversified food products with different sodium contents to meet consumers’ needs for low-sodium products.” Ass 1,2,3, Res4,6,11 and FP 1, 3: “Carrying out in-depth industry investigation and research to clarify the impact of food reformulation to enterprises and consumers and providing technical support for enterprises through various routes to deal with the issues raised in the whole production and sales chain, especially for numerous small and medium-sized enterprises.” [Challenges or barriers to be addressed – Secondary hazards] Res 3,11, Ass 1, 11 and FP 3: “It is not easy to reduce sodium without reducing taste and changes in food properties. This requires enterprises to develop flavor substitutes, use sodium free preservatives, and even change the entire food production process to maintain the water retention rate, yield rate, texture, and antiseptic and bacteriostatic effects of products. These may lead to other health issues.” [Challenges or barriers to be addressed – Compliance and supervision] Res1,9, Cons 1: “Enterprises with high industrial level have better compliance with salt reduction policies based on their relatively high social responsibilities and technical capabilities. However, for small and medium-sized enterprises, they lack the technology and R&D investment to reduce the sodium content of food, and sodium reduction work is not easy to reach them, so their compliance would be relatively poor. This needs more publicity and supervision.”
(4) Publicity	
	[Opportunities] Cons 1,3 and Res 4,10: “China has a relatively mature publicity system and technical team, and various media have many channels of publicity and wide accessibility.” Adm 2, Res 11: “Nutrition and health have gradually become the focus of media publicity. The government (mainly health sector) also carries out salt reduction policy and knowledge publicity with the help of National Lifestyle Month, Salt Reduction Awareness Week and other health days every year.” [Challenges or barriers to be addressed] Cons 1: “The influence of current publicity activities is very limited in improving public awareness and behaviors for sodium reduction. Very few people chose less sodium products when shopping.” FP 2: “Lack of regulatory support which is conducive to the promotion of sodium-reduced products.” Res 1,8: “It is critical to obtain the support of publicity departments outside the health sector through legislation.”
(5) Education and training	
	[Opportunities] Res 6,11: “China has established a preliminary health education and training system, and has relatively mature experience in education, training and material development, which is conducive to the work of education and training on sodium reduction.” [Challenges or barriers to be addressed] Adm 3, Res 8 and Cons 4: “It is lack of training and technical guidance for food producers on food reformulation and for the public on choosing foods with less sodium.” Res 8,9: “We should give full play to the role of various relevant associations and educational institutions in education and training to strengthen the salt reduction awareness of enterprise R&D personnel, improve their food reformulation technology, strengthen national and international communication and technical exchange, and enhance their self-efficacy.” Res 4: “We should actively seek the support and cooperation of the education department to quickly cover the sodium reduction health education to students and their families through school health education courses.”

### General attitudes toward the target-setting policy

In general, two-thirds (18/27) of the participants supported implementing the policy as soon as possible because pre-packaged foods in China usually contain too much sodium and sales are increasing year by year. The other one-third disagreed and thought the contribution of pre-packaged foods to sodium intake was low and that implementing such a policy was not cost-effective (Q1). Differences between stakeholder groups were clear: most supporters were researchers (11/12) and administrators (4/4), and most opponents were food producers (3/3) and association representatives (3/3; Table 2).

Participants in support of a target-setting policy also stated that sodium reduction has been emphasized in national health strategy, consumption of pre-packaged foods among the younger generation is increasing, and the policy will drive food industry innovation and consumer awareness. Meanwhile, those opposed emphasized the importance of consumer awareness and had concerns about impacts on the supply chain and sales, and availability and variety of products (Table 3).

More than half of the participants (15/27), including food producers and those from associations thought enterprises may actively cooperate if they find the sodium contents of some products are too high and might be doing harm to sales, whereas only one participant responded that they did not think enterprises would cooperate and 11 others thought it would be determined by consumer acceptance, government attitude and enterprises’ capacity (Q2; Tables 2, 3).

### Attitudes toward adopting the WHO sodium benchmarks

Nearly all participants (25/27) agreed that the WHO benchmarks and the underpinned food categorization framework should be referred to, but adjustments are needed to fit China’s context. The representative view was that the benchmarks could be taken as long-term goals but would be too ambitious for initial use. To be feasible, the targets should be set step by step based on China’s national conditions including the baseline sodium contents and contribution of individual food categories to sodium intake. (Q3) The majority of participants (18/27) were in favor of choosing a percentile sodium content such as the 75th percentile (Q5) and using an adapted WHO food categorization framework (17/27) to ensure international comparability and applicability for Chinese food products (Q7; Tables 2, 3).

### Key parameters for target-setting

Type of targets: Most preferred only setting maximum sodium targets (13/27) or setting both maximum and mean targets (11/27), although one food producer insisted that the most important thing is to publicize and educate consumers. (Q4) When a mean target was suggested, nearly all respondents (20/21) suggested sales data and market share should be considered (Q6; Table 2).

Priority foods: Regarding implementing the policy, most (20/27) participants preferred setting targets first for the major dietary sodium contributors (Q8; Table 2).

Voluntary or mandatory: A few participants insisted that only mandatory implementation would be effective, but most (23/27) supported voluntary approach. The potential negative impact on enterprise development, lack of readiness for systematic monitoring and evaluation, and gradually transitioning from voluntary to compulsory were the major considerations and suggestions (Q9; Table 2).

Timeline: More than half of the participants (15/27) preferred a timeline consistent with the national goal of “reducing salt intake by 20% by 2030.” However, most association representatives and food producers were more conservative, suggesting that because pre-packaged foods are not the main source of sodium intake, sodium reduction in pre-packaged foods only needs to keep up with the decline in discretionary salt use. Meanwhile, some researchers and administrators suggested that the timeline should be more aggressive considering that China’s target-setting policy has fallen behind and is likely to be implemented on a voluntary basis (Q10; Table 2).

### Opportunities and challenges/barriers to be addressed

In the focus group, opportunities and challenges were more broadly discussed regarding policy, social resources, technical aspects, publicity, and education and training to inform the development of the policy (Q11 and Q12; Table 4).

Most participants believed that the Chinese government is powerful and the policy environment supporting salt reduction has been established. To improve the target-setting policy, the most commonly mentioned challenges/barriers to be addressed were: (1) implementing a comprehensive salt reduction strategy to improve awareness and reduce discretionary salt use simultaneously; (2) strengthening research to clarify the impact of target-setting and provide support to the food industry and stakeholders; (3) engaging social resources in research, education, training and coordination; (4) establishing a performance appraisal and incentive system; (5) maintaining a fair competitive environment; and (6) developing a supportive information system (Table 4).

Most participants suggested that the government should clarify the responsibilities of food associations and societies in leading and supporting their member enterprises in food reformulation. Several key issues were mentioned as needing technical solutions: whether/how to reformulate high-sodium condiments (when less salt added, consumers tend to use/eat more), how to avoid secondary hazards from newly added additives, and how to engage small and medium-sized enterprises. Current publicity activities were regarded as ineffective in encouraging consumers to choose lower-sodium products. It was strongly suggested that legislation would be needed to obtain the support of publicity departments outside the health sector. To improve education and training, it was suggested to empower various relevant associations and educational institutions, and actively seek the support of the education sector to improve the coverage of training and education in a short time (Table 4).

## Discussion

This exploratory study explored the views of key stakeholders on whether and how to reduce sodium in pre-packaged foods through target-setting policy in China, including how to adopt or adapt the WHO global sodium benchmarks. Overall, we found that most participants supported setting sodium targets for pre-packaged foods, with maximum targets set at an agreed percentile based on the current levels of sodium in China’s food supply, and the food categories following the WHO food categorization framework with necessary food supply adaptions. This study findings indicate that setting sodium targets is an important strategy to reduce sodium intake in China. However, there is also a need for strong government leadership and robust monitoring to guarantee the achievement of 20% reduction of sodium content in pre-packaged food by 2030.

### Leveraging the WHO benchmarks

As adopted by the WHO benchmarks, setting maximum sodium targets were supported by most participants because maximum values are the most straightforward concept for governments, consumers and the food industry (24), and allow easy monitoring of foods and companies (25).

Our quantitative study, conducted in parallel with the current study, which modeled the potential impact of the WHO benchmarks alongside other possible targets, revealed the WHO benchmarks would achieve around 50% reduction but were considered too ambitious for initial use. Instead, a set of targets that could lead to a 20% reduction in sodium content from the baseline was preferred (21). This may be a reasonable starting point considering 20% sodium reduction is substantial and may not be noticed by consumers and should be acceptable by most companies (26, 27).

The recent WHO global report on sodium intake reduction noted that 65 of the 194 Member States have implemented a target-setting policy using mandatory (*n* = 21), mandatory and voluntary (*n* = 6), or voluntary (*n* = 38) approaches (3). Mandatory reformulation generally achieves a larger effect than voluntary implementation. Without close government oversight, voluntary targets are not sufficient incentives for the food industry to reformulate; hence, close monitoring and the potential for mandatory targets will be required to ensure the voluntary program has an effect (24). In any case, monitoring of adherence to targets needs to be transparent and independently verified.

### Support across sectors for target setting

While two-thirds of participants supported immediate target setting, there were differences between stakeholder groups. Neither food producers nor representatives of food associations opposed the government’s policy and the public interests on sodium reduction, but they are apparently not ready to support the target-setting policy. As seen in other countries, when considering how to implement such policy, the food industry tends to resist and raise difficulties or challenges (28, 29), neglecting that the existing sodium targets/benchmarks are the evidence that reducing salt/sodium in the food supply is feasible for the food industry and acceptable to consumers (24). Strengthening education, research, training, exchange and technical support, clarifying responsibilities of associations, and promoting legislations for enterprises would be among the most effective solutions. Evidence indicates that sodium reduction by 20–30% for all products is acceptable for most companies (26, 27).

### Combination with other sodium reduction strategies

In China, discretionary salt use dominates the population sodium intake (6, 7). Comprehensive strategies to engage media, families, and catering and food industries must be considered in parallel. To do so, national systematic sodium reduction strategies and promotion mechanisms are needed to ensure multispectral synchronous implementation in a targeted and planned manner. Of course, there are several other options that can complement food reformulation that are not covered in this study, including sales and marketing regulation, improvement of food labeling and taxation of high-sodium foods. WHO is in process of developing guidelines on the use of low-sodium salt substitutes (30), which should be referred to when considering their use.

## Limitations

The study was designed as a very structured and focused survey and discussion, which improved the efficiency of this study but may also have restricted the thoughts of the participants. In addition, the participants were purposively selected and no representatives from other relevant government sectors like education and market supervision were invited. Further extensive interviews, covering researchers and decision-makers from relevant non-health sectors, are meaningful for the formulation and implementation of a target-setting policy for salt reduction.

## Conclusion

Setting sodium targets for different pre-packaged foods was supported by most stakeholders and should be implemented alongside strategies to reduce discretionary salt use during cooking and eating. This study provides information on perspectives, considerations, opportunities, and challenges for a target-setting policy that could be effective in China.

## Data availability statement

The raw data supporting the conclusions of this article will be made available by the authors, without undue reservation.

## Ethics statement

The studies involving humans were approved by National Institute for Nutrition and Health (NINH), Chinese Center for Disease Control and Prevention. The studies were conducted in accordance with the local legislation and institutional requirements. The participants provided their written informed consent to participate in this study.

## Author contributions

PZ and HW conceived the study. PZ, HW, JZ, ER, SP, BZ, GD, XY, and AL contributed to the design of the study. JZ, LD, PZ, and HW collected the data. PZ, JZ, and LD contributed to data analysis. PZ, JZ, HW, ER, and MB drafted the manuscript. ER, SP, RY, FH, MB, CN, and YL critically reviewed the draft manuscript. All authors read and approved the final manuscript.

## Footnotes

^1^ www.wjx.cn
