# Supplementary material for: Setting sodium targets for pre-packaged foods in China — an exploratory study
Source: Front Nutr. 2023 Nov 1;10:1231979. doi: 10.3389/fnut.2023.1231979 (PMC10646300; doi:10.3389/fnut.2023.1231979)
Supplement: Supplementary file 1 [file Table_1.docx]

# Text S1: Guide and questionnaire for the survey and group discussion

- Note 1: The electronic version is available at: https://www.wjx.cn/vj/mBvIgD8.aspx
- Note 2: The objective of the qualitative study was to understand the feasibility of promoting WHO Global sodium benchmarks in China, explore how to leverage the WHO global sodium benchmarks and international experience to help set sodium targets for processed food, and find out acceptable strategies to move forward at present in China.
- Note 3: To help the participants make informative choices and comments, a systematic background introduction was given before the survey and group discussion. The background introduction covered the global experience of target-setting for salt reduction, the WHO global sodium benchmarks for different food categories, the quantitative results of this study, and the current strategies for salt reduction in China. The questionnaire contains 10 multiple-choice or single-choice questions with open questions on certain contents that may be controversial and 3 open ended questions. In addition, there is a completely open question to ask whether there are other opinions or suggestions.

**Key points to be emphasized before the survey and group discussion**

- Excessive sodium intake is the first risk factor in diet leading to life loss of Chinese residents; Reducing salt (sodium) is the most cost-effective strategy to prevent chronic diseases.
- Salt reduction requires a comprehensive strategy. In western countries where processed food is the main source of sodium intake, mandatory labelling for the sodium content of packaged food, setting the target of decreasing sodium content and increasing taxes on high salt food have become the main strategies.
- In China, cooking is still the main way for residents to add sodium. The contribution of processed food to sodium intake is 30.1% or 15.9% if soy sauce and fish sauce are not included. However, with the increasing consumption of processed food, the salt reduction strategy for processed food should also be considered simultaneously, and should adapt to the main salt reduction process, and should not be too aggressive or slow down.
- This study only focuses on the possibility and feasibility of setting sodium content targets for processed foods in China, and other salt reduction strategies are not included in this survey and group discussion.
- To support countries in salt reduction, the World Health Organization released the global benchmark for the highest sodium content of 48 subcategories of foods in 2021. In principle, the benchmark is the lowest of the current target values of all countries, and a special food categorization system has been established for this purpose.
- According to the sodium content and consumption of pre-packaged food in China, if a maximum sodium target for each food subcategory can be set and fully implemented, the estimated sodium content would be reduced by about 6.5% for P90 targets, 20% for P75 targets, 45% for P50 targets, and 50% for the WHO sodium benchmarks if all the subcategories of food achieved targets.

**Outline for the survey and group discussion**

1. **[General view] Which of the following views do you prefer about sodium target-setting for processed foods? (Single choice)**
   1. It is necessary to set appropriate sodium targets for processed foods as soon as possible because the salt content of processed foods in China is too high, and the consumption is increasing year by year.

Additional reasons:

- 1. It is not recommended to implement the target-setting strategy in a hurry because the contribution of processed food to sodium intake of Chinese people is not high (30.1%, including sauces but not including cooking salt), and therefore it is not cost-effective and will cause a waste of resources.

Additional reasons:

1. **[Enterprise’s attitude] What do you think is most likely about the enterprise's attitude towards sodium target setting for processed foods? (Single choice)**
   1. In any case, the food manufacturers will not be willing to cooperate.

Main reasons:

- 1. The food manufacturers may actively cooperate because some products are too salty for most consumers. If the sodium content level of these salty products is limited, it will be beneficial to the sales of the food manufacturers.

Other reasons:

- 1. Others, please specify:

1. **[General opinions on WHO benchmarks] Your opinion on how to learn from the WHO sodium benchmark (including food categorization and benchmarks) is: (single choice)**
   1. The WHO benchmarks draw on the experience of many countries and is recommended to be adopted directly.
   2. It can be used for reference, but it should be adjusted according to China's national conditions.
   3. It is not necessary or impossible to refer to the WHO sodium benchmarks.

Main reasons:

1. **[Types of target] Which sodium target type do you prefer for each category of food? (Single choice)**
   1. Only setting the maximum sodium targets
   2. Setting both the maximum and average sodium targets
   3. Others, please specify:
2. **[Maximum target setting approaches] How do you suggest setting the maximum sodium target for a certain category of food? (Single choice)**
   1. Adopting the WHO sodium benchmarks directly

Main reasons:

- 1. Adopting a percentile level of sodium content in such foods (such as the 90^th^ or the 75^th^ percentile level)

Main reasons:

- 1. Other methods, please specify:

1. **[Average target setting approaches] If you choose (2) in question 4, how do you prefer to set the average sodium targets for the foods in a category? (Single choice)**
   1. A simple average target
   2. An average target weighted by sales or consumption of foods in the category
2. **[Use of WHO food categorization framework] What is your attitude towards the WHO food categorization system used for global sodium benchmarks? (Single choice)**
   1. It is recommended to copy and use, because it is specially established for setting sodium benchmarks, which is relatively reasonable and conducive to international comparison.
   2. It should be referred to as far as possible, but it needs to be supplemented appropriately according to the advantageous products in China.
   3. The WHO system is not suitable for China, so other classification systems should be selected.

Please specify your preferred system:

- 1. I am not an expert in this field and cannot judge which food categorization system is the best.

1. **[Priority food categories] At the initial stage, how to choose the priority food categories to set sodium targets for? (Single choice)**
   1. Based on the average sodium levels of individual food categories, the higher ones are preferred.
   2. Based on the average sodium levels of individual food categories weighted by their sales/consumption shares (i.e. relative contribution to sodium intake), the greater contributors are preferred.
   3. Setting sodium targets for all kinds of food according to certain rules, and there is no need to choose priority food categories.
   4. Others, please specify:
2. **[Mandatory or voluntary] If target-setting strategy will be implemented, do you prefer mandatory or voluntary? (Single choice)**
   1. Voluntary

Main reasons:

- 1. Voluntary first, and transition to mandatory for appropriate categories

Main reasons:

- 1. Mandatory

Main reason:

1. **[Principle of timeline setting] The principle of setting timeline for specific sodium targets: (single choice)**
   1. No need to set an exact timetable because processed food is not the main source of sodium intake.
   2. Should be consistent with the progress of "reducing salt intake by 20% by 2030".
   3. Should be more radical than the progress of "reducing salt intake by 20% by 2030", because the sodium targets might be set for only some products, and it cannot guarantee to meet the targets, especially when adopting voluntary targets.
   4. Others, please specify:
2. **What are the advantages of implementing the target-setting strategy in China in the following aspects?**
   1. Policy and regulation:
   2. Social resources:
   3. Publicity:
   4. Technical aspects:
   5. Education and training:
   6. Other:
3. **What are the challenges and solutions of implementing the target-setting strategy in China in the following aspects?**
   1. Policy and regulation:
   2. Social resources:
   3. Publicity:
   4. Technical aspects:
   5. Education and training:
   6. Other:
4. **Are there any other considerations?**
